# Primary leiomyoma of the ureter: a case report

**DOI:** 10.1186/s13256-021-02960-w

**Published:** 2021-08-03

**Authors:** Shingo Morinaga, Shigeyuki Aoki, Motoi Tobiume, Genya Nishikawa, Hiroyuki Muramatsu, Toyonori Tsuzuki, Reiko Saiki, Junko Hashimoto, Kaori Mori, Yoko Yamaguchi, Akari Kobayashi, Tomoko Sawada, Ruriko Futamachi, Yoshiaki Yamada

**Affiliations:** 1 Department of Urology, Japan Community Health Care Organization, Kani Tono Hospital, 1221-5 Dota, Kani, Gifu 509-0206 Japan; 2 Department of Urology, Japan Organization of Occupational Health and Safety, Asahi Rosai Hospital, 61 Hirakocho, Owariasahi, Aichi 488-8585 Japan; 3 Department of Pathological Diagnostics, Aichi Medical University School of Medicine, 1-1 Karimata, Nagakute, Aichi 480-1195 Japan; 4 Division of Nursing, Japan Community Health Care Organization, Kani Tono Hospital, 1221-5 Dota, Kani, Gifu 509-0206 Japan; 5 Division of Hospital and Clinic Coordination, Japan Community Health Care Organization, Kani Tono Hospital, 1221-5 Dota, Kani, Gifu 509-0206 Japan

**Keywords:** Primary leiomyoma of the ureter, Ureteral neoplasm, Ureteral leiomyoma

## Abstract

**Background:**

Only 14 cases of leiomyoma with ureteral origin have been reported previously. Such primary leiomyomas often present as hydronephrosis, making the diagnosis difficult. Radical nephroureterectomy is often performed because of the possible diagnosis of a malignant tumor. We report the 15th case of primary leiomyoma with a ureteral origin.

**Case presentation:**

A 51-year-old Japanese man presented with a chief complaint of asymptomatic gross hematuria with a history of hypertension. Enhanced computed tomography showed a tumor at the upper part of the right ureter that appeared to be the cause of hydronephrosis and contracted kidney; no retroperitoneal lymphadenopathy and distal metastasis were observed. A well-defined 20-mm (diameter) defect was identified at the upper of the right ureter on retrograde pyelogram with no bladder cancer on cystoscopy. Urine cytology and right divided renal urine cytology findings were negative. Laparoscopic nephroureterectomy was performed, and the extracted tumor measured 20 × 13 mm. Histopathological examination revealed primary leiomyoma with no recurrence 16 months after the operation.

**Conclusions:**

Preoperative examination with the latest available ureteroscopic technology can help preserve renal function in the case of benign tumors by enabling preoperative ureteroscopic biopsy or intraoperative rapid resection. Moreover, nephroureterectomy is recommended in the case of preoperative suspicion of ureteral malignant tumors.

## Background

Primary leiomyoma rarely develops in the urogenital organs. There have been even fewer reports of leiomyoma of ureteral origin. Ureteral leiomyomas often cause hydronephrosis, which make their detection and diagnosis. Therefore, radical nephroureterectomy is often performed owing to the possibility of malignancy.

Our patient also underwent laparoscopic nephroureterectomy for right ureteral cancer. The present report describes this case and includes a review of the relevant reports in literature. To the best of our knowledge, this is the 15th case of primary leiomyoma with a ureteral origin.

## Case presentation

A 51-year-old Japanese man visited our department with a chief complaint of asymptomatic gross hematuria in November 2019 with a history of hypertension. Family history was unremarkable.

At the first visit, height and weight of the patient were 179 cm and 93 kg, respectively. Blood pressure was 128/78 mmHg, pulse was regular with 62 beats/minute. and body temperature was 36.4 °C. The patient had no history of smoking or drinking.

There were no specific abnormalities or neurological findings at the initial physical examination. Blood biochemistry revealed no abnormal findings, and urinalysis revealed microscopic hematuria with red blood cells (RBC) 20–29/high-power field (hpf) and white blood cells (WBC) 1–4/hpf.

**Fig. 1:**
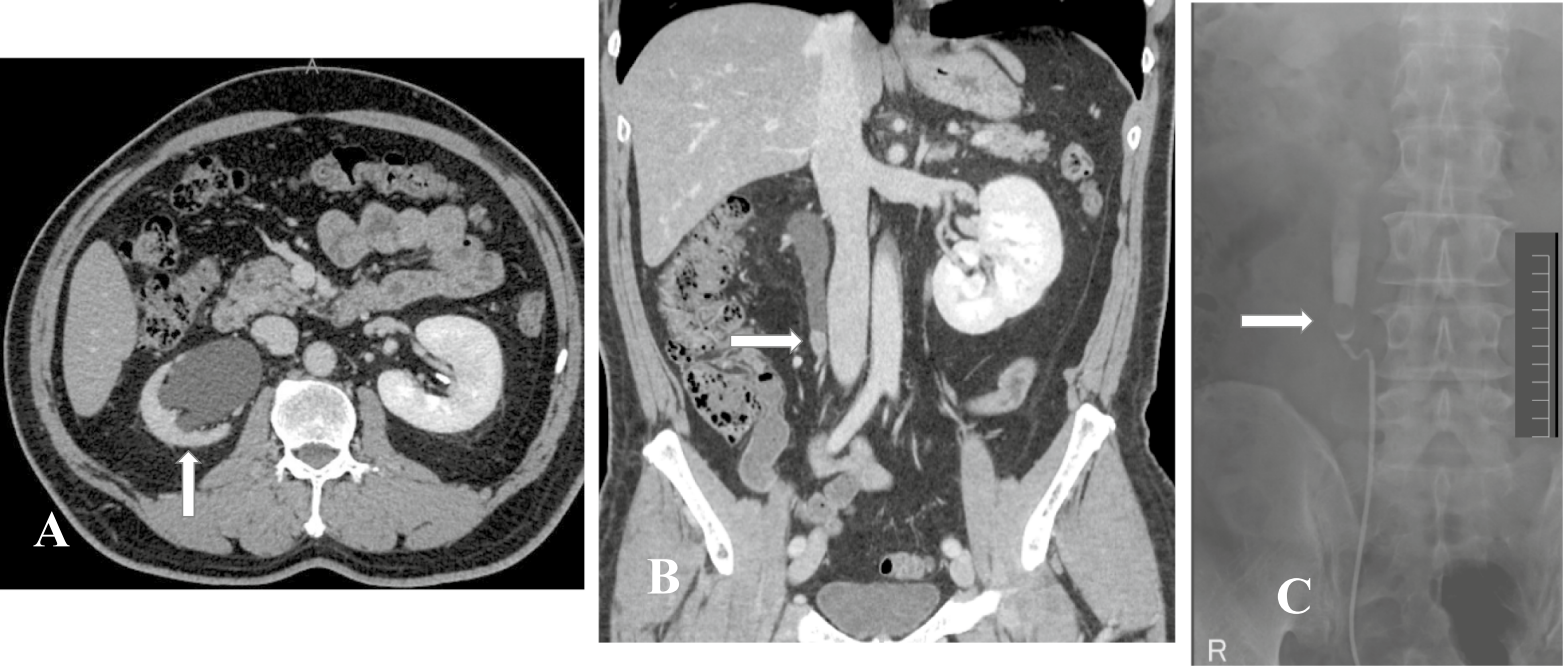
Abdominopelvic enhanced CT showing right ureter with obstruction, contracted kidney, and hydronephrosis (**A**: white arrow). Homogeneous enhanced tumor (**B**: white arrow). A filling defect of approximately 20-mm diameter in the upper right ureter is demonstrated by retrograde pyelogram (**C**: white arrow)

Abdominal computed tomography (CT) revealed hydronephrosis and contracted right kidney, in addition to fatty liver. Enhanced CT showed a tumor in the upper part of the right ureter, with a density similar to the ureteral wall, which appeared to be the cause of hydronephrosis and no retroperitoneal lymphadenopathy (Fig. 1A, B). Distal metastasis was not observed. A well-defined 20-mm (diameter) defect was identified at the upper right ureter on retrograde pyelogram (RP) (Fig. 1C), with no bladder cancer on cystoscopy; therefore, the findings of urinary cytology and right divided renal urine cytology were classified as class I.

Based on the above findings, the patient was diagnosed with contracted kidney with right ureteral cancer (cT2, N0, M0) and right hydronephrosis, and the condition was close to that of a nonfunctional kidney; laparoscopic nephroureterectomy was performed.

**Fig. 2:**
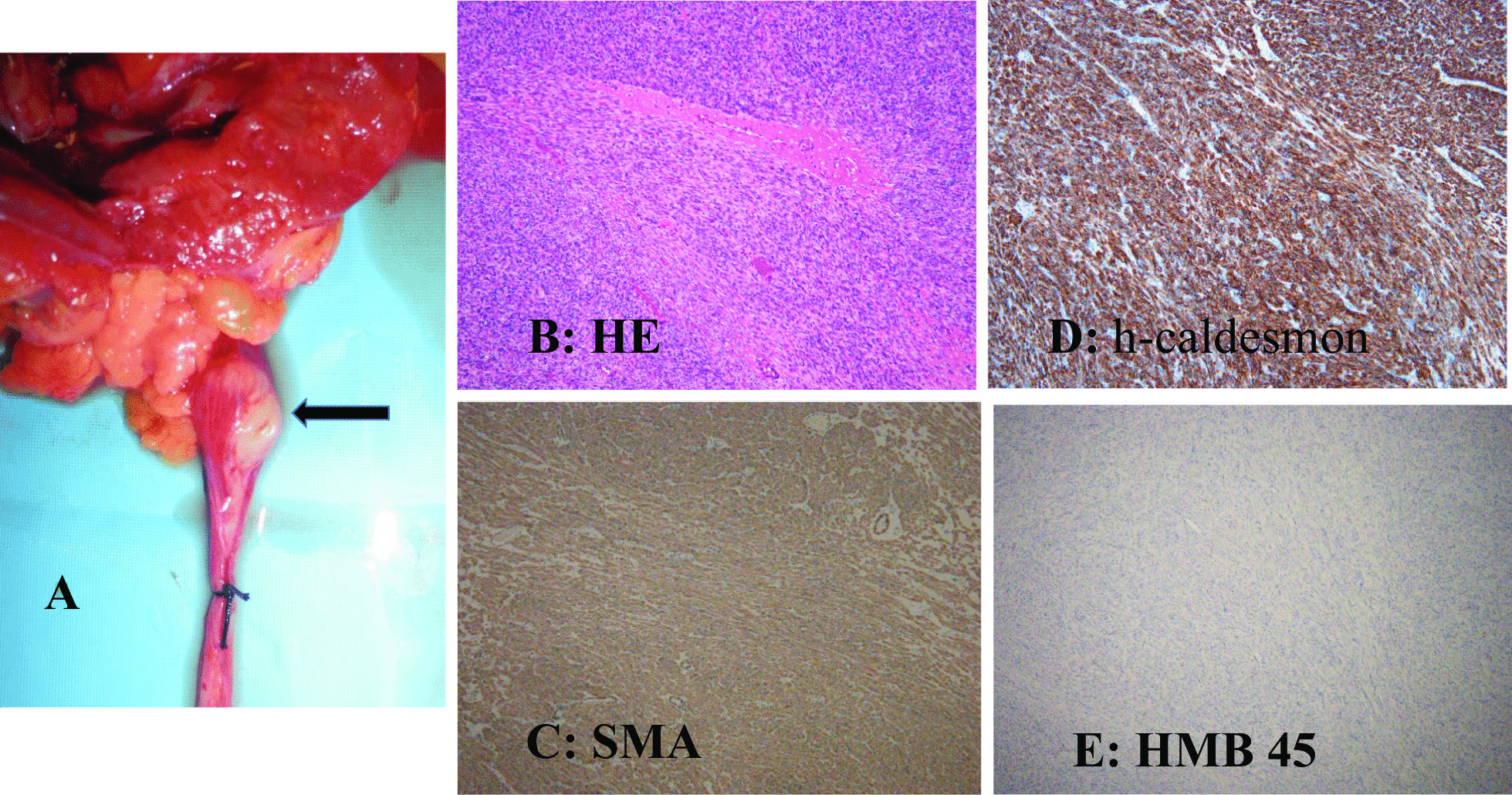
Nephroureterectomy specimen showing a solid tumor measuring 20 × 13 mm with a yellowish->white cut surface (**A**: black arrow). The pathological findings showed interlacing fascicular growth of spindle cells (**B**) [hematoxylin and eosin (HE) ×100]. Immunohistological findings showing positive staining for α-smooth muscle actin (SMA) (**C**) and h-caldesmon (**D**), and negative staining for human melanin black (HMB) 45 (**E**) (×100)

The extracted specimen was a nonpapillary, broad-based, solid tumor measuring 20 × 13 mm with a yellowish-white cut surface (Fig. 2A).

Histopathological findings showed an increase in spindle cells with an interlacing fascicular pattern, mild nuclear atypia, and no mitotic figures (Fig. 2B). Because immunostaining showed α‐smooth muscle actin (SMA) (+) (Fig. 2C), h-caldesmon (+) (Fig. 2D), S-100 (−), C-KIT (−), cytokeratin (−), Melan-A (−), and human melanin black (HMB) 45 (−) (Fig. 2E), it was diagnosed as a primary leiomyoma from the ureteral muscularis.

Neither recurrence nor occurrence of leiomyoma was found in the contralateral urinary tract and other genitourinary organs 16 months after surgery.

## Discussion

Nonepithelial benign ureteral tumors of mesodermal origin are rare, and primary ureteral leiomyomas are even rarer [1]. According to our research, only 14 cases have been reported since the first report published by Leighton *et al*. in 1955 [2–15], making the present patient the 15th (Table 1). Apart from one patient being an infant, most of the patients were aged between 30 and 60 years (average age 41 years), including the present case, aged 51 years. The lesions were located in the right ureter in seven, left in seven, and bilaterally in one. Nine patients were men, and six were women. The site of the lesion was upper, middle, and lower parts in eight, two, and five, respectively. There were no significant differences in the sex, lesion location, and site of development. The tumor sizes of the 12 cases described ranged from 2 to 120 mm (average size 37.5 mm), and were also various.

**Table 1:** Reports of ureteral leiomyoma after 1955

Reference	Age	Sex	Side	Size (mm)	Location	Treatment
Leighton [2]	45	F	Rt	Quite small	Upper	Nephroureterectomy
Kao *et al*. [3]	34	F	Lt	50	Upper	Nephroureterectomy
Mondschin *et al*. [4]	4	M	Rt	20 × 15 × 10	Upper	Nephroureterectomy
Sekar *et al*. [5]	35	F	Rt	Micronodules	Lower	Ureterectomy
Zaitoon [6]	48	M	Lt	25 × 20 × 15	Lower	Partial ureterectomy
Cussenot *et al*. [7]	48	M	Rt	2	Lower	Ureteroscopic biopsy
Igarashi *et al*. [8]	60	M	Lt	10 × 5 × 5	Middle	Nephroureterectomy
Yashi *et al*. [9]	40	F	Rt	7	Upper	Partial ureterectomy
Ikota *et al*. [10]	40	M	Lt	15 × 13 × 12.2	Upper	Nephroureterectomy
Naruse *et al*. [11]	38	M	Rt	35 × 13	Lower	Partial ureterectomy
Nouralizadeh *et al*. [12]	24	M	Lt	120 × 110 × 75	Middle	Partial ureterectomy
Wang HS *et al*. [13]	24	M	Billateral	Unknown	UPJ	Partial ureterectomy
Zehri *et al*. [14]	32	F	Lt	112 × 70 × 15	Upper/middle	Nephroureterectomy
Englund *et al*. [15]	57	F	Lt	36	Lower	Tumor resection
Present case	51	M	Rt	20 × 10	Upper	Nephroureterectomy

UPJ: Ureteropelvic junction

Although the increasing use of laparoscopic morcellation techniques for hysterectomy and management of fibroids has been associated with reports of intraperitoneal seeding and growth of intraperitoneal daughter fibroids [16], and Kho *et al*. described the increased use of laparoscopic treatments for fibroids, with either myomectomy or morcellation of the fibroids, or laparoscopic hysterectomy with morcellation, secondary leiomyoma of the ureter, which grows from the extraureteral wall, may become a more common disease in the coming years [17].

Although inflammation, chronic stimulation, occlusion, and trauma are suspected, the mechanism of primary ureteral leiomyoma development remains unclear. Two patients had a history of ureterolithiasis, but not the present patient. Ikota *et al*. [10] reported a diffuse leiomyoma of the ureter as a complication of multiple endocrine neoplasia (MEN) type 1. In MEN type 1, the complication of multiple leiomyomas is recognized in a variety of organs, including the esophagus, stomach, lungs, uterus, and skin, but this case was the only one to develop leiomyoma in the ureter. It has been suggested that the MEN type 1‐associated gene may have a causal relationship with multiple leiomyoma.

There are no clinical symptoms peculiar to this disease, and many patients are discovered by chance during a detailed examination of other diseases. However, our patient presented with a chief complaint of asymptomatic gross hematuria. The diagnosis is made by diagnostic imaging such as excretory urography, RP or CT, and urinary cytology, as for other ureteral tumors, but there are no characteristic findings.

Differential diagnosis as a benign disease includes perivascular epithelioid cell tumor (PEComa), ureteral polyps, and papilloma, but no characteristic diagnostic imaging method has been established.

Surgical treatment was performed in 14 of the 15 patients. Partial ureterectomy preserving the kidney was performed in only seven patients. Of patients who underwent partial ureterectomy, five were diagnosed by intraoperative rapid section and only two by ureteroscopic biopsy. In the other seven patients, nephroureterectomy was performed without differential diagnosis of benign or malignant tumors before surgery. As our patient was suspected of having cancer in the right ureter with contracted kidney and right hydronephrosis, and the condition was close to that of a nonfunctional kidney, laparoscopic nephroureterectomy was performed.

It has become possible to diagnose benign tumors by preoperative examination as a result of advancements in ureteroscopic technology in recent years. Therefore, if renal function is good and benign tumors are suspected in young people, ureteroscopic biopsy or rapid intraoperative pathology should be performed to make an active histopathological diagnosis, and it is important to pay careful consideration to the preservation of renal function.

## Conclusions

Primary leiomyoma of the genitourinary tract is rare and difficult to diagnose before surgery. Preoperative examination can help preserve renal function in the case of benign tumors by efficient diagnosis and appropriate treatment. Nephroureterectomy in the case of suspicion of ureteral malignant tumor is a logical option.

## Data Availability

Not applicable.

## Abbreviations

CT: Computed tomography
RP: Retrograde pyelogram
SMA: Smooth muscle actin
MEN: Multiple endocrine neoplasia
PEComa: Perivascular epithelioid cell tumor
HMB 45: Human melanin black 45
